# Supplementary material for: MetaRibo-Seq measures translation in microbiomes
Source: Nat Commun. 2020 Jun 29;11:3268. doi: 10.1038/s41467-020-17081-z (PMC7324362; doi:10.1038/s41467-020-17081-z)
Supplement: Supplementary file 10 — Supplementary Data 7 [file 41467_2020_17081_MOESM10_ESM.zip › File2/Confidence_High_Taxonomy/314554_out.krona.html]

Javascript must be enabled to view this page.

members
magnitude
magnitudeUnassigned
count
unassigned
taxon
rank

314554\_out

1

superkingdom
2759
1

kingdom
4751
1

subkingdom
1
451864

1
4890
phylum

subphylum
1
147537

1
4891
class

order
1
4892

family
34353
1

1
4951
genus

species
479094
1

SRS076929\_contig\_number\_14974
